# Exploring the road to public healthcare accessibility: a qualitative study to understand healthcare utilization among hard-to-reach groups in Kerala, India

**DOI:** 10.1186/s12939-024-02191-7

**Published:** 2024-08-09

**Authors:** Surya Surendran, Jaison Joseph, Hari Sankar, Gloria Benny, Devaki Nambiar

**Affiliations:** 1 The George Institute for Global Health, New Delhi, India; 2 Faculty of Medicine, University of New South Wales, Sydney, Australia; 3 Prasanna School of Public Health, Manipal Academy of Higher Education, Manipal, India

**Keywords:** Public healthcare access, Vulnerable groups, Enablers and barriers to healthcare

## Abstract

**Background:**

Kerala, a southern state in India, is known to be atypical due to its high literacy rate and advanced social development indicators. Facing competition from a dominant private healthcare system, recent government health system reforms have focused on providing free, high-quality universal healthcare in the public sector. We carried out an analysis to ascertain the initial impacts of these measures among ‘hard to reach groups’ as part of a larger health policy and systems research study, with a focus on public sector health service utilisation.

**Methods:**

We conducted Focus Group Discussions (FGDs) among identified vulnerable groups across four districts of Kerala between March and August of 2022. The FGDs explored community perspectives on the use of public healthcare facilities including enablers and barriers to healthcare access. Transliterated English transcripts were coded using ATLAS.ti software and thematically analyzed using the AAAQ framework, supplemented with inductive code generation.

**Results:**

A total of 34 FGDs were conducted. Availability and cost-effectiveness were major reasons for choosing public healthcare, with the availability of public insurance in inpatient facilities influencing this preference. However, accessibility of public sector facilities posed challenges due to long journeys and queues. Uneven roads and the non-availability of public transport further restricted access. Gaps in acceptability were also observed: participants noted the need for the availability of special treatments available, reduced waiting times for special groups like those from tribal communities or the elderly mindful of their relatively greater travel and need for prompt care. Although quality improvements resulting from health reform measures were acknowledged, participants articulated the need for further enhancements in the availability and accessibility of services so as to make public healthcare systems truly acceptable.

**Conclusion:**

The ‘Kerala Model of Development’ has been applauded internationally for its success in recent years. However, this has not inured the state from the typical barriers to public sector health care use articulated by participants in the study, which match global evidence. In order to deepen the impact of public sector reforms, the state must try to meet service user expectations– especially among those left behind. This requires attention to quality, timeliness, outreach and physical access. Longer term impacts of these reforms – as we move to a post-COVID scenario - should also be evaluated.

**Supplementary Information:**

The online version contains supplementary material available at 10.1186/s12939-024-02191-7.

## Introduction

Kerala, a southern state in India, is known to be an atypical state in the country due to its high literacy rate and advanced social development indicators [1–3]. Compared to the majority of Indian states, Kerala has continually been a notable exception with better health outcomes and indicators, particularly in relation to Reproductive, Maternal, Newborn, and Child Health (RMNCH) [4]. This has been possible through the priority given by the state government over the years towards improving health infrastructure, with a special focus on primary care [5]. The number of Primary Health Centres (PHCs) in the state increased from 369 in 1960 to 1356 in 2004 [6]. Kerala invested in infrastructure to create a multilayered health system designed to provide basic services in the community and expanded primary health care coverage for the prevention and treatment of diseases [7]. Various policies have been implemented, especially at the primary level, to improve access to public health care with the more recent one being the Aardram mission [8, 9]. The Aardram mission was designed to transform existing PHCs into Family Health Centres (FHCs) providing enhanced facilities and services with an objective to make healthcare more accessible [9, 10].

The government, from the time of formation of the state, has placed emphasis on social(ized) welfare, and development. It significantly increased the number of public healthcare institutions between 1961 and 1986 [11]. The 1982 National Health Policy promoted national regulations that set in motion widespread privatization of healthcare. This led to rapid growth of private institutes ranging from small clinics to high-end institutes having several specialties to cater to in-patient services [11, 12]. This was a major shift in the healthcare sector, with private institutes continuously improving the quality of care provided so as to stay in the competition. As the private sector bloomed at the cusp of India’s neoliberal turn in the early 1990s, the public healthcare system began to decline [13]. Compared to other states, currently, the presence of private healthcare sector in Kerala is high and plays a significant role in the state’s overall healthcare system [14, 15]. The private healthcare sector in Kerala dominates in terms of the number of doctors, hospitals, and beds, making it the area with the highest concentration of healthcare facilities in India [13]. As a result, out-of-pocket expenditure of people across different class groups has increased [13].

Seeking care at a private health facility is expensive. Poor people in Kerala have been unable to afford treatment at private facilities [16], and catastrophic health expenditure has been on the rise [17], leading to increased inequities and medical impoverishment [12]. With the 1992 decentralization reforms, local self-government bodies were given control over public healthcare facilities with the intention of improving performance [16]. Reforms focused on improving treatment and access, highlighting the need for equitable coverage [18]. Multiple improvements had to be brought into the healthcare system including the quality of care provided, and the human and clinical resources within the system. The year 2012 was a milestone year in the state, marking a formal foray into Universal Health Coverage reforms, with an emphasis on upgradation of primary care [19]. Building on this momentum, in 2017, the Aardam mission was introduced by the incoming government to transform the public healthcare system and provide comprehensive services at the grassroots level [20].

Marginalized groups, especially the poor, face disadvantages and often rely on the public health system for their needs [21]. In addition to the financial disadvantage, these groups have restricted access to healthcare due to their gender, age, social status, geographical habitat, physical disabilities, etc. Because they frequently face discrimination, there is a need to exercise measures that prevent them from being exploited [22]. They are at a disadvantage or ‘left behind’ as compared to others, primarily as a result of their restricted access to medical treatment and the key health determinants, such as clean and hygienic drinking water, nutrition, accommodation, and sanitation [2]. This is in part why, on the path to UHC, particular emphasis has been placed on health equity and ‘leaving no one behind’ [21, 23, 24].

In India, the National Health Policy of 2017 makes similar pronouncements, calling for equitable and affordable healthcare to all citizens of India, regardless of their socioeconomic status [25]. Exclusion is a major challenge within and beyond the health sector in India: women, people from minority castes, and from tribal communities have historically faced disadvantage and social exclusion, which has restricted their access to healthcare and education over time [26]. Reforms like Aardram have implicitly sought to improve health care access and outcomes in groups such as these. In the context of various reform efforts, it is imperative to assess their impact on vulnerable groups in order to ascertain whether the intended beneficiaries are indeed receiving the benefits of these reforms. Therefore, as part of a larger health equity study, we explored the perspectives from the left-behind groups on their health seeking behaviours, and the use of public health facilities.

## Methods

### Study setting and design

We carried out a qualitative study using Focus Group Discussions (FGDs) in four districts of Kerala, India: Trivandrum, Kollam, Alappuzha and Kasaragod. The districts were chosen using random sampling method [27]: two primary healthcare facilities were chosen in each district and the area (panchayat) these facilities served were chosen as study sites.

### Participants

Vulnerable groups at the study sites were identified through previous phase of data collection where secondary analysis of household data was carried out to identify groups left behind, and the local leaders and healthcare workers were interviewed about health system reforms [28]. Communities identified in our quantitative analysis as well as through the interviews common among all districts were people from Scheduled Caste backgrounds, Scheduled Tribes^1^, women, the elderly, and palliative care patients. Previous analyses as well as our extensive site visits also pointed towards geographic-specific groups such as fisherfolk, persons affected by Endosulfan exposure and those living with particular disabilities.

### Data collection

FGDs were conducted by a team of two female (SS and GB) and two male researchers (JJ, HS) led by a senior researcher (DN) between March and August 2022. A topic guide was developed by the researchers and implemented after initial pilot testing and later tailored according to the type of participant groups in each FGD (Topic guide attached as Supplementary File 1). The questions in the topic guide prompted the participants to speak about their healthcare experiences with various facilities that they usually visit. In addition to the tool, any further topics that came up during the discussion were further explored.

Initial permissions were taken from local and district-level authorities. Potential participant groups were identified and organized for discussion with the help of healthcare workers from the primary healthcare facility and local self-government and community leaders. We also contacted NGOs to identify vulnerable groups and for their recruitment. The discussion was conducted at a time and place convenient for the participants. While not completely successful, efforts were made to avoid any form of gatekeeping from the system actors, and any such influence identified was excluded from the transcript during analysis. When the group assembled, detailed information about the purpose of the study, identity of the researchers, and the role of participants were given by the researchers and clarifications required, if any was provided before proceeding towards data collection. Individual verbal consent was taken from all participants included in the audio recording.

All the FGDs were conducted in Malayalam, the local language in the state, and in Hindi with inter-state migrants. The discussions were audio recorded with the permission of participants and stored in a secure local server. The audio recordings were outsourced to a transcription company to transliterate verbatim to English, after signing a confidentiality agreement. Each English translation was then cross-checked by the researchers for fidelity to the original recordings in Malayalam.

### Data analysis

A thematic analysis approach was used to analyse the data [29]. The data was coded line by line using ATLAS.ti software [30]. An initial set of codes and code groups were formed using the topic guide and one transcript which was discussed among the researchers. The code book was modified based on inputs from team members and finalized for further coding. Regular discussions were held to discuss the codes that emerged through additional analysis. For the purpose of this paper, codes relevant to data on the drivers and barriers to the use of public healthcare facilities and participant narratives of utilizing them were extracted and analyzed. We used the Availability, Accessibility, Acceptability and Quality (AAAQ) framework [31] to understand the various enablers and barriers to seeking healthcare at public facilities.

### Ethics

Ethics approval was granted by the Institutional Ethics Committee of the George Institute for Global Health (Project Number 05/2019). Informed consent was taken from the participants and their confidentiality maintained. Only researchers had access to the participants’ identifying information.

## Results

We conducted 34 FGDs/group interviews^2^ including participants from diverse backgrounds residing in facility catchment areas across four districts in Kerala. The number of participants ranged between 5 and 16 with a majority having 6–8 participants. We tried to segregate the participants by age and gender to make them homogenous, but some groups could not be age and gender-matched due to a lack of turnout/loss to follow up between recruitment and data collection. Table 1 shows the distribution of participant groups across the four districts.

**Table 1:** Participating groups details

FOCUS GROUP DISCUSSIONS (*N* = 34)					
	**Thiruvananthapuram**	**Kollam**	**Alappuzha**	**Kasaragod**	**Total**
People from Scheduled Tribe	1	-	1	2	**4**
People from Scheduled Caste	3***	1	2	1	**6**
Palliative care patient carers	1	1	-	1	**3**
Elderly	1	1	1	2	**5**
Fisherfolk	-	2	2	-	**4**
Migrant workers	-	-	2	1	**3**
Organised sector workers (coir ^*^; tobacco)	-	2	-	1	**3**
Mahatma Gandhi National Rural Employment Guarantee Act (MGNREGA) workers^**^	2	2	1	-	**5**
Total	**8**	**9**	**9**	**8**	**34**

^*^Coir factory workers are individuals who are involved in the traditional process of making ropes from coconut fibers [32] and bidi workers are individuals who work in the process of rolling raw tobacco into dried leaves to create bidi, a locally used smokable tobacco [33]. They are typically employed in small-scale industries where their wages are dependent on the amount of output they produce.

^**^MGNREGA (Mahatma Gandhi Rural Employment Guarantee Act) is an Act brought by the Government of India to provide 100 days of wage employment to households in rural areas [34]. They were identified as a “vulnerable” population because they heavily depend on the program for income, receiving minimal wages that are significantly below market rates, exposing them to economic insecurity.

^***^One of our Focus Group Discussions with Scheduled Caste males likely included local males who were not from this social group, but rather from privileged castes

We detail first, the healthcare-seeking preferences of participants; and second, the enablers and barriers that influenced their care-seeking at public facilities.

### Preference of healthcare facility

Preference of health facility was governed by type of care needed, the distance and ease of transport to the facility, availability of infrastructure, quality of care, as well as a number of other factors like existing access to social security, insurance and other health-related schemes. These are presented as enablers in the following section.

**Fig. 1:**
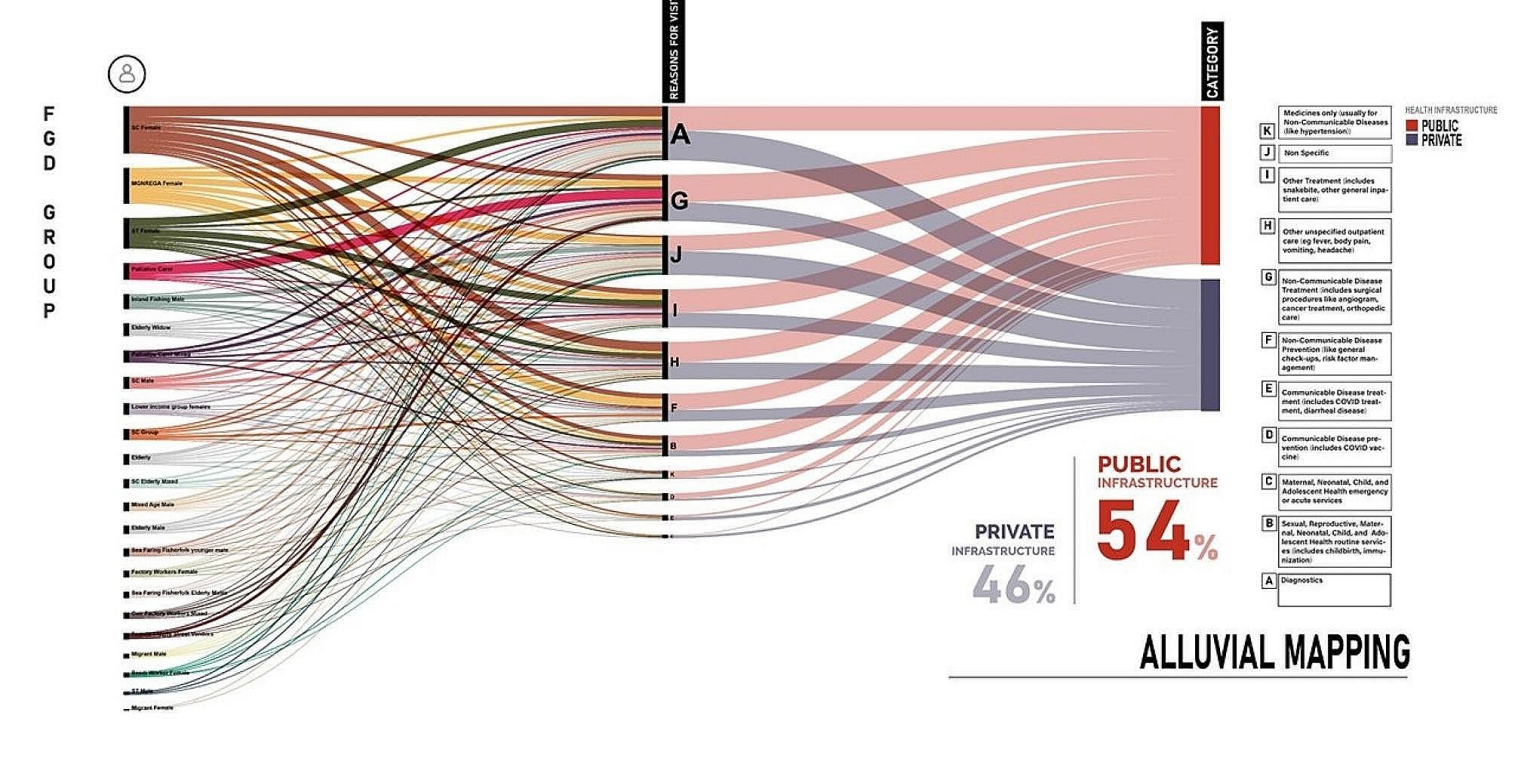
Alluvial map showing the reasons to visit health facilities by sector for hard-to-reach groups across the four study sites

As part of our parent study, we conducted a quantitative analysis of the discussions within FGDs/ group interviews, specifically focusing on individuals’ mentions of their healthcare-seeking behavior for past illness. These mentions were further categorized based on the type of groups and the nature of illnesses discussed, and the results were visually represented on an alluvial map (Fig. 1). Although public healthcare facilities were the preferred choice in over half of the cases, the variation was relatively minimal. The preference for a government facility was for general non-acute ailments such as fever or cold. In times of emergencies or when the need for in-patient care arose, myriad options was considered, with variations between and within the groups: tertiary level of government facility (e.g., medical college) or private hospitals being common choices. Newly upgraded FHCs were commonly utilized for management of long term Non-Communicable Diseases (NCDs), where medication needs were continuous. We found some mention on the utilization of FHC in almost every group with variation in how they were frequented. Some preferred going to a secondary facility (Community Health Centre-CHC, Taluk or district hospital) even for routine consultations. At the time of our fieldwork, Sub-Health Centres (SHC) were rarely utilized and in general, a higher facility was preferred within the government health system even if similar services were available at the lower level. Diagnostic services were an exception: private facilities were approached because of the quick turnaround time for the procedure and receiving the results. The choice of healthcare also depended on the perquisites people had through employment or within their households (like social security, insurance, enrollment in palliative care programs). Migrants in the organized sector we interviewed exclusively consulted a private nursing home which had a tie up with the factory they were working for and provided free healthcare services. Some of the participants had Employee State Insurance (ESI) which covered their and their family’s health expenses at an ESI operated hospital or dispensary.

…the surgery was done in a private hospital. They don’t need money. We gave the papers of ESI and thus they didn’t take any money…. My son works at a private company and there he had ESI card – SC male, Alappuzha.

Sometimes, even though unaffordable, private health facilities were used and expenses were met by borrowing money or pawning gold.

I was admitted in a private hospital in Palakkad for treatment of leg swelling due to diabetes…It costed me around Rs 1.5 lakh! (USD 1880)^5^… Lives matter, right? So we borrowed money for the treatment.- ST female, Alappuzha

Apart from these, there were condition-specifical preferences which prompted people to choose a healthcare facility based on the illness that they were facing. In such cases, by virtue of non-availability of services, their usual preferred facility was disregarded.

We go to X FHC for common illness… I went to RCC [the Regional Cancer Centre] in Trivandrum for my cancer treatment. – Beedi roller female, Kasargod.

### Barriers and enablers of public health facility use

We laid out barriers and enablers of public health facility use using the AAAQ framework.

### Availability

*The range of services available at primary centres was limited, leading to bypassing to higher levels or private sector usage*.

There was a mixed opinion among participants on the availability of services in public healthcare facilities, particularly FHCs and sub centres which by design offered fewer services. Participants felt more services should be offered at the FHC or sub centre level.

If someone here gets a fever, there is no doctor [at sub centre] whom we can consult. So there is no option for us, but to go to CHC or medical college… If this sub centre had any sort of development, then we could go there if there is a need. If there is a doctor, we could have consulted them. But that is not there. They only give care to pregnant women and conduct immunization drives. That too, only sometimes a week. It does not function most times in a week. – Fisherman, Alappuzha.

Considering the fact that higher level of facility meant a wider range of services, the participants bypassed FHCs and sub-centres for CHC or district level hospitals, even if the former were closer to their homes. This saved them the effort and time of travel in case a referral was required, especially for conditions which could be fatal.

Since there are chances that after going to CHC, we will be referred to Medical College, instead of wasting time, we go there directly. – MNREGA female, Alappuzha.

Even when FHCs were the first choice of treatment, in times of emergencies, a private facility was preferred, particularly during the evening and night hours when FHC was not functional.

A drawback with the hospital is that during night there is absence of facility for all [acute] diseases. So, people are made to depend on nearby private hospitals during the night. -Elderly male, Kasaragod.

Medicines were constantly referred to as being unavailable at public facilities. In a Thiruvananthapuram FGD with Scheduled Caste women, one noted:

Medicines are never available there and I have to always buy from outside. Only paracetamol is available there. That’s why we are consulting private hospitals. It’s not because we are financially capable to go there, but we have to go there if we want a quick cure… Expensive medicines won’t be there.- SC female, Trivandrum.

### Accessibility

*Physical inaccessibility was a barrier to public service utilization.*

Even though an FHC was planned to service all members in the panchayat, all communities could not access FHCs due to their location. While within walking distance for some people, others had to travel long distances, change buses, or pay large amounts to hire autorickshaws that would drop them at FHCs. Therefore, even if the care received was free, the additional cost incurred in the process of reaching there made it unaffordable.

In some areas, a higher-level facility was closer than the FHC. For example, all groups in one facility area of Kasaragod mentioned that they preferred going to the CHC, which was only a short distance away from their home and had direct bus access. An elderly person in Kasaragod explained:

I only go to CHC. The government hospital I used to and still go to is CHC. There is no [direct] bus to FHC. We won’t get a bus. It’s difficult. -Elderly mixed, Kasaragod.

In other cases, where a public healthcare facility was unavailable close by, private clinics and hospitals were depended upon, which were closer to home.

There is no travel facility to Perumon [FHC]. We can go there only by auto. It cost over Rs. 100. Another hundred for coming back. So, it will cost Rs.200 for the whole trip; that is the problem… hence we go to private hospital which is nearby. – Elderly men, Kollam.

In times of emergencies, a facility in close proximity was preferred. Since many private hospitals with in-patient facilities were located in areas with good road and transport access, they were the default choice.

We prefer private hospitals in emergency situations. There was this incident when she cut her hand and because the blood was flowing, and the nearest hospital was [name of the private hospital]. We usually prefer the nearby hospital and after dressing the wound, if they recommend us to visit the government hospital, then we’ll go there. -Coir factory workers female, Kollam.

Autos were the most commonly used means of transport. They were seen to be relatively cheap and provide the comfort of travel which would not be possible through the public bus where one may or may not get a seat during travel.

The moment that we get a hint of physical ailment coming up, we would have an auto-rickshaw at our doorstep. We would rush to the hospital. In most houses, there is an autorickshaw. Regardless of night or day, an auto would be available. Our houses are situated on the roadside, just like where we are right now, and they would take us to the CHC or wherever we need for treatment.- MNREGA female, Alappuzha.

Buses were considered unreliable due to their unavailability at the required hour. This was especially true in remote areas where bus thoroughfare would be sparse and infrequent. Hence, people relied on autos which could be summoned at any time. They were considered more expensive than bus travel but economical when compared to other available options, like taxis.

Even for taking an X-ray, we have to travel the same distance. If the total cost [of treatment] is Rs.1000, around Rs. 800 will be for travel charges alone. We use the bus rarely; we use it mainly if we are going to the OP. There is a bus at 9 am [but] if we have to reach there on time, we will have to go in an auto. If we go by bus, we will be late. There will be a rush. So, we call an auto- ST male, Thiruvananthapuram.

Some communities felt cut off from access to healthcare itself because of where they were located geographically. The SC community in Thiruvananthapuram lived on a hill which was not fully connected by road; therefore, the sick had to be carried by fellow members until the point where there was road access. Similarly, the SC community in Alappuzha lived in flood prone area which made commuting difficult, especially during the monsoon season. Tribal communities in Thiruvananthapuram were sparsely distributed in forest areas: the closest health facility was 15 km away and they end up spending Rs 600 (USD 7.5)^6^ for the commute.

During rainy season, the roads are flooded. Maybe they will find a way to carry the ill person if an emergency arises. The rain is not light nor is the water feeble, it is harsh and heavy. There was a situation where the waterflow was so heavy that our vehicle could not even move. We had to wait for hours [until the water level decreased]. -MNREGA female, Thiruvananthapuram.

*Financial accessibility (i.e. Affordability) was an enabler of public sector utilization, enhanced by use of publicly funded insurance*.

The most common reason for choosing a public health facility was the availability of care for a very low or no expense from the people’s side. Since public healthcare facilities are funded by the state, the out-of-pocket expenditure for patients was relatively very low with some services costing less than 1% of what was charged by private hospitals.

My wife had cancer and was taken to a private hospital. She required radiation and chemotherapy they said it would cost Rs. 25000 and Rs. 75000 (USD 313 and USD 940)^4^ for each respectively. We could not afford it so they suggested we go to a public hospital where it would be cheaper. The next day we went to General Hospital where my niece arranged everything and they started the radiation right away. Each radiation cost only Rs 50 (USD 0.6)^4^ and the chemotherapy was done for free. They did 23 radiations and 5 sessions of chemotherapy- SC male, Alappuzha.

Most services were free or offered at a nominal charge. For example, a blood test at an FHC cost around Rs.15 (USD 0.18)^6^, an amount which almost equalled the minimum charge for travelling on a public bus.

I go to [name of the CHC] hospital for sugar test, it will be open till noon. Post-noon it is closed. The charge is Rs. 10 (USD 0.12)^4^. Whereas in a private clinic, they took around Rs. 90 (USD 1.1)^4^ from me. The same Rs. 10 test is Rs. 90 there. -ST female, Kasaragod.

In addition to the almost free healthcare facilities, the state also provided public insurance to the poorest of the poor. This could be used while availing in patient services at any public healthcare facility. Having an insurance, however, meant that their choice of healthcare facility was restricted by that. The recipients of public insurance were forced to use public facilities (and some private facilities where it was accepted) for treatment. Therefore, they preferred to go to a public facility even if they were not happy with the treatment there.

I had surgery for Hernia at General hospital… Only expense was for the medicines that we bought from outside. As we were having the [insurance] card, other [expenses] were provided by the hospital itself. -SC male, Alappuzha.

*Information inaccessibility was a barrier to public sector utilization*.

As aforementioned, the government was providing insurance for poor populations which covered any in-patient care service availed by them. These insurance schemes were available at all in-patient government facilities. While these insurance schemes were valid for services in some private hospitals, there was relative lack of clarity on this.

We can use the public insurance card only for impatient treatment, what about for other things? We have received an [insurance] card from the health department. But where to use it… we are not sure- ST female, Kasaragod.

Information on services was not widespread in groups we spoke with. Many participant groups reported that they never had any awareness sessions from the health system. Occupational groups that had stronger ties to LSG and already availing of government entitlements, like MGNREGA and organized sector workers, were aware and had better access to these facilities while other groups like the fisherfolk were not.

Last month, the JHI [Junior Health Inspector] and others came took a class on TB. -Beedi worker female, Kasaragod.

Although social media was used to spread and receive updates on medical services, there was no system in place for regular updates.

Getting treatment at a public hospital involved running around to different departments and it was noted in an FGD that knowing someone from inside the hospital would help facilitate the process-

They will make us go in circles [vattam karakum]. My father’s sister’s daughter was the duty nurse there. And so because we knew someone there… -SC male, Alappuzha.

### Acceptability

*Special treatment was an enabler of public sector utilization, while language was a barrier for some*.

Some of the groups mentioned during FGDs that they received preferential treatment at the FHC because of their “vulnerability status”. This positive discrimination allowed the participants to avail healthcare at a faster rate as they could jump the queue. These groups included palliative caretakers, the elderly who could not wait for a long time, and the tribal community who needed to travel long distances to reach the FHC.

For migrants who came to Kerala from other language-speaking states (mostly the North Indian states), conversing in Malayalam and letting the doctor know their issue was a challenge. However, in Alappuzha, at the private nursing home where they were treated as per company policy, the migrants were provided with Hindi-speaking doctors and staff; therefore, enabling conversations between them.

Additionally, even though doctors in the public health facilities were forbidden from practicing privately, some doctors had clinics at their home and to receive proper treatment at the hospital, the patients were required to consult the doctors with a fee at their private clinic. These instances were recalled with dissatisfaction by participants.

### Quality

*Higher perceived quality of private sector, alongside long waiting times and treatment delays in the public sector were barriers to public sector utilization*.

There was a mixed opinion on the quality of services provided by public facilities. Tertiary hospitals were considered to have good healthcare service: perceived quality reduced as the facility level lowered. It was felt that FHCs and SHCs did not have the infrastructure and resources for providing treatment. Participants expressed experiences of being referred from one hospital to the other for lack of services, hence thought it is best to directly visit a higher-level facility.

Since there are chances that after going to [name of CHC], we will be referred to [name of public Medical College], instead of wasting time, we go there directly – MNREGA female, Alappuzha.

There was a general perception that the basic quality of services was adequate in private facilities, even with (or perhaps because of) higher costs. This was a major reason that participants reported use of private facilities even though it was not considered affordable. As a fisherman in Kollam said:

If we go to private hospitals, even if the cost is high, we will get adequate care in time. -Fisherfolk male, Kollam.

Participants who went to private hospitals chose it because the turnaround time for consulting and treatment was less when compared to public facility.

Whatever reason we say, government hospitals are the best. But the drawback is that we have to wait a lot. In private hospitals, nurses will come [take care of us quickly] since it is a business. -Elderly male, Kollam.

Government health facilities catered to a large number of patients in the area. As the level of the facility increased, the number of patients seeking treatment also increased. Since people visited secondary and tertiary level hospitals even for small ailments, the waiting time also correspondingly increased. People had to wait hours to get a doctor’s appointment, days and weeks to get scanning done and months before their surgery could be scheduled. Getting a doctor’s consultation meant spending a day at the hospital: people had to miss out on work with possible loss of pay. This was seen as an inconvenience and therefore private hospitals were chosen to get the consultation done in an hour or two after which participants could go on with their regular life.

We have to wait in the queue in the early morning. The OP starts at 8 o’clock but we have to set off at 6:30 or 7 o clock in the morning if we are going from here, then only we can get in firstly in the queue. After consulting the doctor and buying medicine… Some of the doctors are only available till afternoon but OP doctors may be available till evening. We have to depart from here at between 6:30 am and 7:00 am in the morning, wait to take the OP ticket and then wait in the queue for the medicines. Medicine is available at two to three points. The thing is that we will have set off in the morning itself– - ST female, Alappuzha.

The waiting time to consult at public facilities was reported to be too long as there are more patients and fewer doctors. Even for a minor illness, participants had to spend the whole day at the facility waiting for their turn to be treated.

The problem is that it takes a lot of time [at Taluk hospital]. Because there are a lot of people, we have to wait for a long time in the OP itself. The hospital functions based on a token system. We go in the morning and take a token and have to wait till our turn comes. So it will take a whole day. Sometimes it’ll be 2 pm when we are called. Effectively a whole day will be spent there. If we arrive there a bit late, we can only come back by 4pm. If an emergency case comes, the doctor will go to attend it. We will have to wait for the doctor to come back. Since we don’t have to pay any money, everyone will wait there patiently. -2 Lower income group females, Thiruvananthapuram.

Diagnostic services in public healthcare facilities were delayed- sometimes the scheduled date for testing would go beyond a month time. Therefore, even though doctors from a public facility would be consulted, the diagnostics are usually done at private centres so as to not waste time. It was noted that many privately owned diagnostic centres have been set up outside major tertiary care public hospitals.

In the medical college if there is a rush, we would be given dates to come and take a scan… It can be after 1–2 weeks, or after days… So instead of wasting time, we would resort to private institutions- If we spare some Rs. 500 or so, we could get our fix from a private establishment.- 2 fisherfolk male, Alappuzha.

At a micro level, the competence and compassion of individual staff members determined why they preferred a particular facility. Based on experience and hearsay, the quality of the doctor was concluded and if they were good, the facility was visited irrespective of other barriers

A hospital like this is a blessing for this place. All the nurses standing there and all the field workers are available whenever we need any help. I have a bedridden patient and I myself have sugar and pressure. They look after all that although’they don’t have to do it. -Palliative caretakers, Kollam.

The availability of quality services including consulting, diagnostics and medicines at a single facility was considered favorable for the participants. While it was acceptable to have diagnostics and medicines not available at the facility, when it was coupled with improper treatment at the facility, participants chose to go to a private facility instead even though it would be very costly.

[In the public hospital], while examining us they don’t check us with their stethoscope, they just only ask about the illness while sitting on the other side and they prescribe medicine on the basis of the thing we said to them. They don’t examine us properly. Also, if we need to test our blood, most of the time the lab is closed and when we give it for testing, they ask us to come back one or two days later. So we take a test from a private lab, get the result and consult the doctor about it the next day. That’s why we are dependent upon private institutions. Whatever it is, we have to go to private labs, so it’s better to go to a private hospital because everything is available there. The only difficulty is you need money for everything. Everything will get done in half an hour or an hour. -2 ST female, Alappuzha.

One caveat applies. With the Aardram mission of health reforms in place, there were some positive changes noted by participants in the FHC, which reinstated hope for a better service at public facilities.

Things are not like before. There is a lot of change. Doctors will ask us in detail about our problems and explain the reasons. They will tell us that a particular medicine is not in stock, so they will prescribe a different medicine. They don’t prescribe medicines from outside, they prescribe what is available and tell us that will also be effective and to continue that. Now things have changed drastically.- Elderly male, Kollam.

## Discussion

Our analysis sought to characterize the experiences and opinions of the ‘hard-to-reach’ groups on the utilization of public healthcare institutes in Kerala. We saw that their preference of a healthcare facility depended on a range of factors like cost and distance. The limited range of services available at primary centres often led individuals to seek care at higher-level public facilities or in the private sector. Physical inaccessibility served as a significant barrier to accessing public services, while financial accessibility, particularly affordability and the presence of publicly funded insurance facilitated the use of public healthcare. India’s national public health insurance scheme, the Pradhan Mantri Ayushman Bharat Jan Arogya Yojana (PM-JAY) provides cover for in-patient care on a family floater basis and has high public sector empanelment of facilities [35]. In Kerala, our findings suggest that public sector empanelment has incentivized the usage of government services. Information inaccessibility presented challenges to accessing public services. Furthermore, some individuals were encouraged to use the public sector due to the perception of receiving special treatment, while language barriers deterred others. Notably, a preference for the perceived higher quality of private healthcare, coupled with concerns about long waiting times and treatment delays in the public sector, acted as barriers to its utilization.

The recently published India brief from People’s Voice Survey showed 25% of people using public hospitals as their usual source of care and 24% for private, while others did not have a preference [36]. We also found that their preference changed as per need. For example, even though public institutes were chosen for small or chronic ailments, in times of emergencies, private health facilities were used, even if expensive. This preference was probably due to the lack of proper emergency care services in the state, as highlighted in a recently published systematic review [15].

We found that the different enablers and barriers as reflected through the AAAQ framework were influencing each other. The participants often preferred higher level facilities, even for minor illness because of their close proximity and a general perception that services were not available at lower-level facilities. This meant there was a greater patient load at high-level facilities, which would in turn result in long waiting times and treatment delays, another barrier to use of public health facilities reported by the participants.

In order to reduce the delay in treatment, in addition to increasing the health staff as recommended by the participants, the focus also needs to be on improving lower-level facilities such that the visits to public healthcare facility are redistributed. This has been seen in a number of studies in India and globally on bypassing of primary care [37–39]. A study conducted in Northern India found that he ability of healthcare providers to deliver quality clinical care had a more significant impact in reducing bypassing than the physical conditions of PHC facilities, such as building maintenance and availability of medication [39]. In our study, the non-availability of doctors at the SHC prompted people to use higher facilities like the FHC and CHC.

Although the public sector provided medical healthcare at free or nominal cost, expenses still emerged. Even after availing public health insurance, patients incurred expenses related to drugs and diagnostics that were not available – as seen in other studies [40]- as well as transportation costs.

Insurance coverage also affected utilization. Results from a systematic review indicate a positive effect of insurance on healthcare utilization in India, but no clear evidence yet on reducing OOP expenditures or increasing financial risk protection [41]. Our findings suggest that even though the public facilities or empaneled private facilities were preferred as a result of the availability of public insurance, the additional costs associated with hospitalization, such as transport and food, still posed a financial burden on patients. A recent publication highlighted that there was 20% higher healthcare expenses among members covered under the public insurance as compared to nonmembers which can be attributed to their preference for private hospitals over public hospitals [41]. While insurance schemes offered coverage, it still left members with significant out-of-pocket expenses. This unintended effect of health coverage resulted in members spending more on healthcare rather than less [41].

Many reported incomplete information on the schemes and services provided by the public sector. This is an important finding because while literacy levels in Kerala are high, it appears health literacy may not be. Gaps in health literacy have been found in India and the region, and stand in the way of effective coverage and service utilization in both the public and private sector [42]. The use of WhatsApp for dissemination of information has been growing, especially since the COVID 19 pandemic hit [43, 44]. With the rampant access and recent attempts by local stakeholders to improve digital literacy [45], there is a potential for official dissemination of information through social media and improving patient information [46]. This could help bridge the gap of knowledge between those with closer relations with and access to government services and those who are more distant. A study conducted in central and Eastern India found that awareness of public welfare schemes (not including insurance) was greater among self help group members [47]. Therefore, Kerala’s thriving *Kudumbashree* self help group programme could be a launch-pad to promote health literacy; this is an area warranting further exploration.

Apart from tackling just the clinical assessment and treatment of diseases, there is a need to look at the determinants of health, ensuring everyone has access to them [48]. We saw that the inequity in access to health was further impeded by their access to proper infrastructure such as roads, etc. The socially excluded groups have been seen to live remotely in hills or disconnected to the roads. Tribal groups have traditionally inhabited hill tract areas [49], or sea-faring fisherfolk who live on the coast are often cut off from the main centre, thereby impeding their access to timely healthcare intervention. Even though access was considered a major issue among the groups, this did not come through in the recommendation for specific occupational groups like fisherfolk or factory workers. This could be because their occupation itself provided them the mobility that was not available for the other groups- partnerships with employers can play a major role in helping address healthcare needs, although such arrangements are less clear in the informal sector. Further, even some formal sector groups faced barriers that were particular to the group which obstructed their use of public healthcare facilities. For example, migrants working in the formal sector in Alappuzha did not like going to the public facility as providers there could not communicate in Hindi. Further studies looking at particular groups in-depth could understand the difference in needs which could have been missed in our study.

## Conclusion

The ‘Kerala Model of Development’ has been applauded internationally for its success in the recent years. While it leads in the public healthcare activities, there still exist various barriers to accessing healthcare at public funded facilities. Our study highlights the complex and interrelated factors influencing the utilization of public healthcare institutes by ‘hard to reach’ groups in Kerala. The AAAQ framework revealed that enablers and barriers were influencing each other, with higher-level facilities being preferred for minor ailments due to their availability and perception of limited services at lower-level facilities. Gaps in health literacy, particularly among socially excluded groups, hindered effective coverage and service utilization. To address these challenges, there is a need to focus on improving lower-level facilities, promoting health literacy through digital channels and community-based programs such as the Kudumbashree self-help group program. In its mission to provide free public healthcare of quality, the State must look at the various challenges faced by marginalized groups in accessing healthcare, to ensure no one is left behind. Our findings call for an integrated change not just within the healthcare system but also associated facilitators to access such as the public transport, calling for a multisectoral plan for addressing healthcare challenges to ensure equitable access to healthcare services for all.

### Electronic supplementary material

Below is the link to the electronic supplementary material.

**Supplementary Material 1**: Focus Group Discussion Topic Guide.

**Supplementary Material 2**: Abstract in Malayalam.

## Data Availability

The datasets used during the current study are available from the corresponding author on reasonable request.

## Footnotes

1 Scheduled Caste and Scheduled Tribe groups are recognized by the Indian constitution to have been marginalized in the society.

2 Although we set out to conduct FGDs, we conducted 21 group interviews instead of FGDs as fewer than 8 persons were present at the time of interview.

5 INR value converted to USD based on exchange rate in year 2022.

6 INR value converted to USD based on exchange rate in year 2022.
