# Supplementary material for: Exploring the road to public healthcare accessibility: a qualitative study to understand healthcare utilization among hard-to-reach groups in Kerala, India
Source: Int J Equity Health. 2024 Aug 9;23:157. doi: 10.1186/s12939-024-02191-7 (PMC11312678; doi:10.1186/s12939-024-02191-7)
Supplement: Supplementary file 1 — Supplementary Material 1: Focus Group Discussion Topic Guide. [file 12939_2024_2191_MOESM1_ESM.docx]

**Assessing Equity of Universal Health Coverage in India – Focus Group Discussion Topic Guide**

*While waiting for all to arrive, start creating a map (of SHC) and doing a general ice-breakers*

*Explain purpose, and introduce ourselves. Then ask all to introduce themselves.*

*Go through details of consent form and ask if any questions. Share consent forms and indicate option to give verbal consent.*

*Switch on recording and say you give consent, pass on to participants*

*We have made a map of this area and need your help completing it so we can understand things like where you seek health care and other social services. We are putting this map up here, we need a volunteer to be our teacher and add to this.*

***Other colleague to take note of what is not being covered***

1. **Services map**
   1. *Let us start drawing. Where are we? What are the main roads here?*
   2. *What are other main geographic features (water bodies, wildlife)*
   3. *What are the main meeting points/offices here (Kudumbashree, panchayat office, post office, etc.) What are other main landmarks?*
   4. *Where are the main health facilities or hospitals? (SHC, labs, pharmacies/chemists, also PHCs -if off the map, indicate distance and main way to get there from where we are)*
   5. *What are the other places that can help you get/stay healthy? (playgrounds, walking areas, gyms, stadiums)*
   6. *Where is there ambulance access?*
   7. *For which areas/populations is access generally difficult? (can we mark this on the map?)*

***Any questions to add from my colleague?***

1. ***Patient journeys***
   1. *We have mapped all these areas. Could you describe experiences of seeking care in these places?*
      1. *For what condition*
      2. *How do you get there?*
      3. *What is the cost?*
      4. *Is this place accessible to everyone here? Is some group being left out?*
   2. *(if not covered) Let’s start with the SHC –do people in this group go to the SHC?*
      1. *For what conditions (if not, why not)*
      2. *How do you get there?*
      3. *What is the cost?*
      4. *Is this place accessible to everyone here? Is some group being left out?*
   3. *Now onto the FHC – do people in this group go to the FHC?*
      1. *For what conditions (if not, why not)*
      2. *How do you get there?*
      3. *What is the cost?*
      4. *Is this place accessible to everyone here? Is some group being left out?*
   4. *Let’s look at another facility – a private one –*
      1. *For what conditions (if not, why not)*
      2. *How do you get there?*
      3. *What is the cost?*
      4. *Is this place accessible to everyone here? Is some group being left out?*
   5. *What about scans/xrays or tests – where do people go?*
      1. *For what conditions*
      2. *How do you get there?*
      3. *What is the cost?*
      4. *Is this place accessible to everyone here? Is some group being left out?*
   6. *And medicines -where do people get them*
      1. *For what conditions (if not, why not)*
      2. *How do you get there?*
      3. *What is the cost?*
      4. *Is this place accessible to everyone here? Is some group being left out? Why? In what way*
   7. *What are other barriers or challenges to care that are not on here? (eg. animals, lockdowns, seasons, dogbite, attitude or providers, etc.)*

***Any questions to add from my colleague?***

***Let us break for tea 🡪 Team regroups to check flow/follow-up***

*Just a few follow up questions on what we were discussing earlier*

- 1. *In addition to going to facilities, there may be services or schemes that come to your house or area? Could you describe these?*
  2. *Where do you get health related information?*
  3. *We had mentioned health burdens and where you go to get care for them earlier. Has there been a health situation where you were not able to seek care? Or you felt helpless? If yes, what happened?*

1. ***Health determinants, consequences***
   1. *For the health challenges we have already discussed, why do you think they have come up? What are the causes or determinants?*
   2. *Also for the challenges you mentioned, how have they impacted your daily lives? (Probe: expenses, ability to work, caregiving responsibilities)*
2. ***Well-being and suggestions***
   1. *We’ve already talked about what causes health problems. What would you say produces or gives health? Makes someone healthy*
   2. *Who is responsible for making this happen? What is the role of government?*
   3. *We wish to help improve how the local health facility in your area cares for you. Help us come up with a list of things that things can be improved. (probe Who should be responsible for these changes VHSNC, Ward-level committees, ASHA?*
