# Supplementary material for: Exploring the road to public healthcare accessibility: a qualitative study to understand healthcare utilization among hard-to-reach groups in Kerala, India
Source: Int J Equity Health. 2024 Aug 9;23:157. doi: 10.1186/s12939-024-02191-7 (PMC11312678; doi:10.1186/s12939-024-02191-7)
Supplement: Supplementary file 2 — Supplementary Material 2: Abstract in Malayalam. [file 12939_2024_2191_MOESM2_ESM.docx]

**പൊതുജനാരോഗ്യ പരിപാലന ലഭ്യതയിലേക്കുള്ള വഴി:: പാർശ്വവത്കരിക്കപ്പെട്ട വി**ഭാ**ങ്ങളിലെ ജനങ്ങളുടെ പൊതുജനാരോഗ്യ മേഖല സേവനങ്ങളുടെ ഉപയോഗം മനസിലാക്കാൻ ദക്ഷിണേന്ത്യൻ സംസ്ഥാനമായ കേരളത്തിൽ നടത്തിയ ക്വാലിറ്റേറ്റിവ് പഠനം**

**സംഗ്രഹം**

**പശ്ചാത്തലം**

ഉയർന്ന സാക്ഷരതാ നിരക്കും നൂതന സാമൂഹിക വികസന സൂചകങ്ങളും കാരണം ഇന്ത്യയിലെ ഒരു ദക്ഷിണേന്ത്യൻ സംസ്ഥാനമായ കേരളം മറ്റു സംസ്ഥാനങ്ങളിൽ നിന്നും വ്യത്യസ്തമായി അറിയപ്പെടുന്നു. പ്രബലരായ സ്വകാര്യാ മേഖല ആശുപത്രികളോട് മത്സരിക്കുന്ന പൊതുജനാരോഗ്യ മേഖല, സമീപകാല നവീകരണ പദ്ധതികളിലൂടെ പൊതുജനാരോഗ്യമേഖലയിൽ സൗജന്യവും ഉയർന്ന നിലവാരമുള്ളതുമായ സാർവത്രിക ആരോഗ്യ പരിരക്ഷ നൽകുന്നതിൽ ശ്രദ്ധ കേന്ദ്രീകരിച്ചു. ഈ നവീകരണങ്ങളുടെ പ്രഥമമായ അനന്തരഫലം വിശകലനം ചെയ്യന്നതിനായി പാർശ്വവത്കരിക്കപ്പെട്ട വിഭാഗങ്ങളിലെ ജനങ്ങളുടെ ഇടയിൽ പൊതുമേഖലാ ആരോഗ്യ സേവന വിനിയോഗത്തിനു ഊന്നൽ നൽകി പഠനം നടത്തി.

**പഠന രീതി**

ഈ പഠനത്തിന്റെ ഭാഗമായി പൊതുജനങ്ങളുടെ അഭിപ്രായം അറിയുന്നതിനായി കേരളത്തിലെ നാലു ജില്ലകളിൽ നിന്നും പാർശ്വവത്കരിക്കപ്പെട്ട വിഭാഗങ്ങളിലെ ജനങ്ങളുമായി 2022 മാർച്ച് മുതൽ ഓഗസ്റ്റ് വരെയുള്ള കാലയളവിനുള്ളിൽ ഫോക്കസ് ഗ്രൂപ്പ് ചർച്ചകൾ (എഫ്ജിഡി) നടത്തി. ഈ ചർച്ചകളിൽ ഓരോ മേഖലയിലുമുള്ള ടി വിഭാഗങ്ങളുടെ പൊതുജനാരോഗ്യ സേവന ലഭ്യത, ഉപയോഗം, ആരോഗ്യപരിചരണം മികച്ചതാക്കിയ ഘടകങ്ങൾ, പരിചരണം ദുഷ്കരമാക്കിയ ഘടകങ്ങൾ തുടങ്ങിയവ സമഗ്രമായി പഠന വിധേയമാക്കി. പരിഭാഷപ്പെടുത്തിയ ഇംഗ്ലീഷ് ട്രാൻസ്ക്രിപ്റ്റുകൾ ATLAS.ti സോഫ്റ്റ്വെയർ ഉപയോഗിച്ച് കോഡ് ചെയ്യുകയും ഇൻഡക്റ്റീവ് കോഡ് ജനറേഷനോടൊപ്പം AAAQ ചട്ടക്കൂട് ഉപയോഗിച്ച് പ്രമേയപരമായി വിശകലനം ചെയ്യുകയും ചെയ്തു.

**കണ്ടെത്തലുകൾ**

മൊത്തം 33 എഫ്ജിഡികളാണ് നടത്തിയത്. സർക്കാർ ആശുപത്രികളുടെ ലഭ്യതയും, ആരോഗ്യ പരിചരണത്തിനുള്ള കുറഞ്ഞ ചിലവും ആണ് പൊതുജനാരോഗ്യ മേഖലയിലുള്ള ആശുപത്രികൾ ആരോഗ്യ സേവനങ്ങൾക്കായി തിരഞ്ഞെടുക്കാനുള്ള പ്രധാന കാര്യങ്ങൾ, അതോടൊപ്പം സർക്കാർ ആരോഗ്യ ഇൻഷുറൻസ് വഴിയുള്ള സൗജന്യ കിടത്തി ചികിത്സയുടെ ലഭ്യതയും ടി ആശുപത്രികളെ ആരോഗ്യ പരിചരണത്തിനായി തിരഞ്ഞെടുക്കാൻ സ്വാധിനിച്ചു. എന്നിരുന്നാലും, ആരോഗ്യ സേവനം ലഭിക്കുന്നതിന് ദീർഘ ദുരം യാത്ര ചെയ്യേണ്ടതും, ആശപത്രികളിലെ ക്യൂവും പരിചരണം ലഭിക്കുന്നതിനുള്ള വെല്ലുവിളികളായി. അസന്തുലിതമായ റോഡുകളും പൊതുഗതാഗതം ലഭ്യമല്ലാത്തതും ആരോഗ്യ പരിചരണം തേടുന്നതിനെ കൂടുതൽ പരിമിതപ്പെടുത്തി. ആരോഗ്യ സേവനങ്ങളുടെ സ്വീകാര്യതയിലെ വിടവുകളിൽ സ്പെഷ്യലിറ്റി ചികിത്സയുടെ ലഭ്യത കൂട്ടുക, ഗോത്ര സമൂഹങ്ങളിൽ നിന്നുള്ളവർ അല്ലെങ്കിൽ പ്രായമായവർ തുടങ്ങിയ പ്രത്യേക വിഭാഗങ്ങളുടെ ആശുപത്രിയിലെ കാത്തിരിപ്പ് സമയം കുറയ്ക്കേണ്ടതിന്റെ ആവശ്യകത തുടങ്ങിയവയാണ്. ആരോഗ്യ പരിഷ്കരണ നടപടികളുടെ ഫലമായുള്ള ഗുണനിലവാര മെച്ചപ്പെടുത്തലുകൾ അംഗീകരിക്കപ്പെട്ടിട്ടുണ്ടെങ്കിലും, പൊതു ആരോഗ്യസംരക്ഷണ സംവിധാനങ്ങൾ ശരിക്കും സ്വീകാര്യമാക്കുന്നതിന് സേവനങ്ങളുടെ ലഭ്യതയിലും, സാർവത്രിക പ്രവേശനം നൽകുന്നതിനും കൂടുതൽ മെച്ചപ്പെടുത്തലുകളുടെ ആവശ്യകത പഠനത്തിൽ പങ്കെടുത്തവർ ഉയർത്തിക്കാട്ടി.

**ഉപസംഹാരം**

'കേരള മാതൃകയിലുള്ള വികസനം' സമീപ വർഷങ്ങളിൽ അന്താരാഷ്ട്ര തലത്തിൽ പ്രശംസ പിടിച്ചുപറ്റിയിട്ടുണ്ട്. എന്നിരുന്നാലും, പഠനത്തിൽ പങ്കെടുത്തവർ വ്യക്തമാക്കിയ പൊതുമേഖലയിൽ നിന്നും ആരോഗ്യ പരിചരണം തേടുന്നതിന് ദുഷ്കരമാക്കിയ സാധാരണ ഘടകങ്ങളിൽ നിന്ന് സംസ്ഥാനത്തിന് കരകയറാൻ ആയിട്ടില്ല. ഇത് ആഗോള തെളിവുകളുമായി പൊരുത്തപ്പെടുന്നു. പൊതുമേഖലാ ആരോഗ്യ സംവിധാനങ്ങളുടെ നവീകരണങ്ങളുടെ സുശക്തഫലം വർദ്ധിപ്പിക്കുന്നതിന്, സേവന ഉപയോക്താക്കളുടെ പ്രതീക്ഷകൾ നിറവേറ്റാൻ സംസ്ഥാനം ശ്രമിക്കണം - പ്രത്യേകിച്ചും സാമൂഹികമായി പിന്നോക്കം നിൽക്കുന്ന വിഭാഗങ്ങളുടെ. ഇതിന് ഗുണനിലവാരം, സമയബന്ധിതത, ഔട്ട്റീച്ച്, ആശുപത്രീകളുടെ ഭൗതിക ലഭ്യത എന്നിവയിൽ ശ്രദ്ധ ആവശ്യമാണ്. കോവിഡിന് ശേഷമുള്ള സാഹചര്യത്തിലേക്ക് നീങ്ങുമ്പോൾ ഈ പരിഷ്കാരങ്ങളുടെ ദീർഘകാല പ്രത്യാഘാതങ്ങളും വിലയിരുത്തണം.
